# Silencing long noncoding RNA NEAT1 alleviates acute liver failure via the EZH2-mediated microRNA-139/PUMA axis

**DOI:** 10.18632/aging.202927

**Published:** 2021-04-26

**Authors:** Qiang Wang, Shu Liu, Huan Wang, Lian Liu, Sheng Zhang, Yingzi Ming, Yujun Zhao, Ke Cheng

**Affiliations:** 1 Transplantation Center, The Third Xiangya Hospital of Central South University, Changsha 410013, P.R. China

**Keywords:** acute liver failure, long noncoding RNA nuclear enriched abundant transcript 1, microRNA-139, p53-upregulated modulator of apoptosis, methylation

## Abstract

This study aimed to investigate the role of long noncoding RNA (lncRNA) nuclear-enriched abundant transcript 1 (NEAT1) in the development of ALF. We collected blood samples from patients with acute liver failure (ALF) and established an ALF mouse model induced by D-galactosamine/Lipopolysaccharide (D-GalN/LPS) for *in vivo* studies. Peripheral blood mononuclear cells (PMBCs) induced with LPS were isolated for *in vitro* experiments. Survival tests, histological analysis, and biochemical indicator assays were conducted. Luciferase assay was performed to determine the binding affinity between microRNA-139 (miR-139) and p53-upregulated modulator of apoptosis (PUMA). Expression of lncRNA NEAT1, enhancer of zeste homolog 2 (EZH2), and PUMA was upregulated, while the expression of miR-139 was downregulated in clinical samples and D-GalN/LPS induced ALF mouse model. LncRNA NEAT1 promoted the enrichment of H3K27me3 on the promoter region of miR-139 *via* EZH2, which led to suppression of miR-139. The inhibition of miR-139 resulted in the upregulation of its downstream target PUMA. The NEAT1/miR-139/PUMA pathway upregulated the production of pro-inflammatory cytokines, tumor necrosis factor alpha, interleukin (IL)-6, and IL-1β, thereby mediating the progression of ALF. In conclusion, silencing lncRNA NEAT1 upregulated the expression of miR-139 through EZH2, leading to the downregulation of PUMA, which alleviated the development of ALF.

## INTRODUCTION

Acute liver failure (ALF) is a rare but life-threatening liver disorder, which is associated with the rapid onset of clinical morbidity and high mortality [1]. Ever since the advent of emergency of liver transplantation, the overall survival rate of ALF has increased to about 70% and the 2-year survival rate reaches 92.4% [2]. Aside from liver transplantation, currently available treatment modalities for ALF include hepatocyte transplantation, extracorporeal liver support devices, molecular adsorbent recirculating system, plasmapheresis and bioartificial liver support systems [3]. Key features of ALF include a systemic inflammatory response, abnormal coagulation, and upregulation of aminotransferases activity including Alanine aminotransferase (ALT) and Aspartate transaminase (AST) [4]. A rapid development of the condition typically results in widespread necrosis and apoptosis of hepatocytes and can lead to multi-organ dysfunction within a few days [5]. In ALF, patients without preexisting hepatic diseases may suffer an acute loss of liver tissues with only hepatic stromal framework remaining and impaired liver regeneration further complicates recovery [6]. Etiological factors of ALF include viral infections such as hepatitis B, autoimmunity, drug toxicity and ischemia [7]. Since the exact cause of severity of ALF and the molecular basis behind the development of ALF remain unclear, effective therapeutics are still being investigated.

Long noncoding RNAs (LncRNAs) comprise a class of RNAs that are over 200 nucleotides in length but cannot be translated into protein [8]. LncRNAs are emerging as important regulators of gene expression, cell proliferation and differentiation [9]. In particular, LncRNA Nuclear-enriched abundant transcript 1 (NEAT1) has been noted as an oncogenic mediator and was found associated with hepatocellular carcinoma [10]. Another study demonstrated that lncRNA NEAT1 promoted the development of lipopolysaccharide (LPS)-induced liver injury [11] and inflammatory response *via* the activation of inflammasomes [12]. Other investigators noted that in glioblastoma, lncRNA NEAT1 bound to the histone methyltransferase enhancer of zeste homolog 2 (EZH2) and subsequently inhibited the expression of its downstream target genes [13]. EZH2 has been found to upregulate the expression of microRNA-139 (miR-139) in pancreatic carcinoma by promoting the tri-methylation of H2K27 [14]. Currently, the molecular functions of lncRNA NEAT1 in human diseases are considered as a subject of significant research interest.

miRNAs are conserved small RNA, ranging from 22 to 25 nucleotides in length and located throughout the human genome [15]. The 3’ untranslated region (3’-UTR) of targeted mRNA is recognized by miRNA and degraded through the formation of RNA-induced silencing complexes [16]. Notably, in ageing research, miRNAs in plasma, muscle, and mitochondria have been found as significant contributors to frailty pathophysiology, leading to physical dysfunction and disability [17]. In particular, miRNAs have shown therapeutic value in hearing loss related to age [18]. In ALF, the application of microarray and sequencing techniques has unraveled novel information regarding the alteration and function of miRNAs in ALF disease models [19, 20]. miR-139 has been reported as downregulated in herb-induced liver injury [21] and hepatocellular carcinoma [22], indicating that miR-139 plays a physiological function in maintaining hepatic homeostasis. However, the detailed mechanisms of miR-139 involvement in ALF have not been elucidated. Here, we enrolled samples of patients with ALF and also developed an ALF murine model to explore the regulatory roles of lncRNA NEAT1 and lncRNA NEAT1-mediated miR-139/p53-upregulated modulator of apoptosis (PUMA) pathway in ALF, which can reveal novel putative molecular therapeutic targets.

## RESULTS

### D-GalN/LPS induced ALF mouse model is established successfully

**Figure 1:**
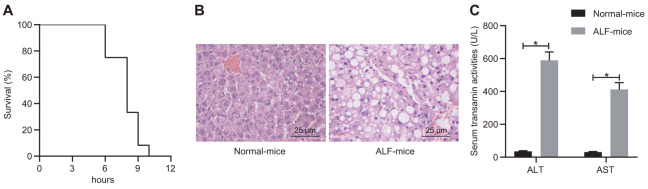
**D-GalN/LPS induced ALF in the mouse model.** (**A**) Survival curve of mice receiving i.p. injection of D-GalN and LPS within 12 h. (**B**) Histological analysis of liver tissues with H&E staining. (**C**) Biochemical analysis to detect the activity of ALT and ALF in serum samples collected from ALF mice and control mice. Data were summarized as mean ± S.D. from at least 3 independent biological replicates. * indicates *p* < 0.05. Data were compared using the unpaired student’s *t*-test. n = 12 mice for each group.

We first utilized the combination of D-GalN and LPS to induce an ALF mouse model. In response to D-GalN and LPS, a progressive increase in lethality was observed between 6 - 12 h post-injection (Figure 1A). ALF is accompanied by liver dysfunction and hepatocytes necrosis [23], so we analyzed biochemical indicators in mouse serum and histology of liver tissues at 6 h after D-GalN/LPS induction. H&E staining analysis showed obvious disordered hepatic structure, along with expanded and bruised liver sinusoids in ALF mice, as opposed to normal mice (Figure 1B). Hepatocyte necrosis and apoptosis were observed in the presence of D-GalN/LPS (Figure 1B). Biochemical analysis revealed significant upregulation in the activity of ALT and AST in ALF mice compared with control mice (Figure 1C). These results suggest the successful induction of ALF using D-GalN/LPS.

### LncRNA NEAT1 is upregulated in ALF

**Figure 2:**
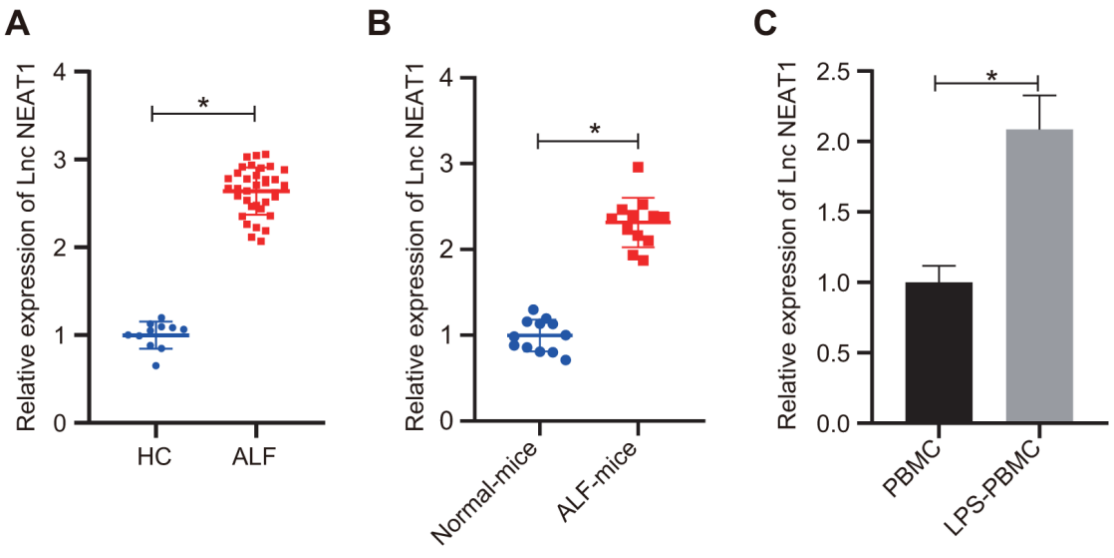
**LncRNA NEAT1 was upregulated in ALF.** (**A**) RT-qPCR was used to analyze the lncRNA NEAT1 levels in human serum samples. Healthy control (HC): n = 11. ALF: n = 35. (**B**) The expression levels of lncRNA NEAT1 was determined by RT-qPCR in liver tissues from control mice and D-GalN/LPS-induced ALF mice. (**C**) Results of RT-qPCR for determining the lncRNA NEAT1 level in human PBMCs with or without 10 ng/mL LPS treatment. The expression level of lncRNA NEAT1 was normalized to the control groups. Data were summarized as mean ± S.D. from at least 3 independent biological replicates and * indicates *p* < 0.05. Data were compared using the unpaired student’s *t*-test.

To further investigate the mechanism regulating the liver failure, we detected the expression levels of lncRNA NEAT1 in patient serum samples with ALF and D-GalN/LPS-induced ALF murine liver tissues. The results showed that the expression levels of lncRNA NEAT1 in both patients with ALF (Figure 2A) and ALF mice (Figure 2B) were significantly increased as compared with that in control groups. Furthermore, LPS treatment induced significant upregulation of lncRNA NEAT1 in human PBMCs *in vitro* (Figure 2C). These data demonstrated the upregulation of lncRNA NEAT1 in ALF conditions.

### Silencing lncRNA NEAT1 alleviates the occurrence of ALF

**Figure 3:**
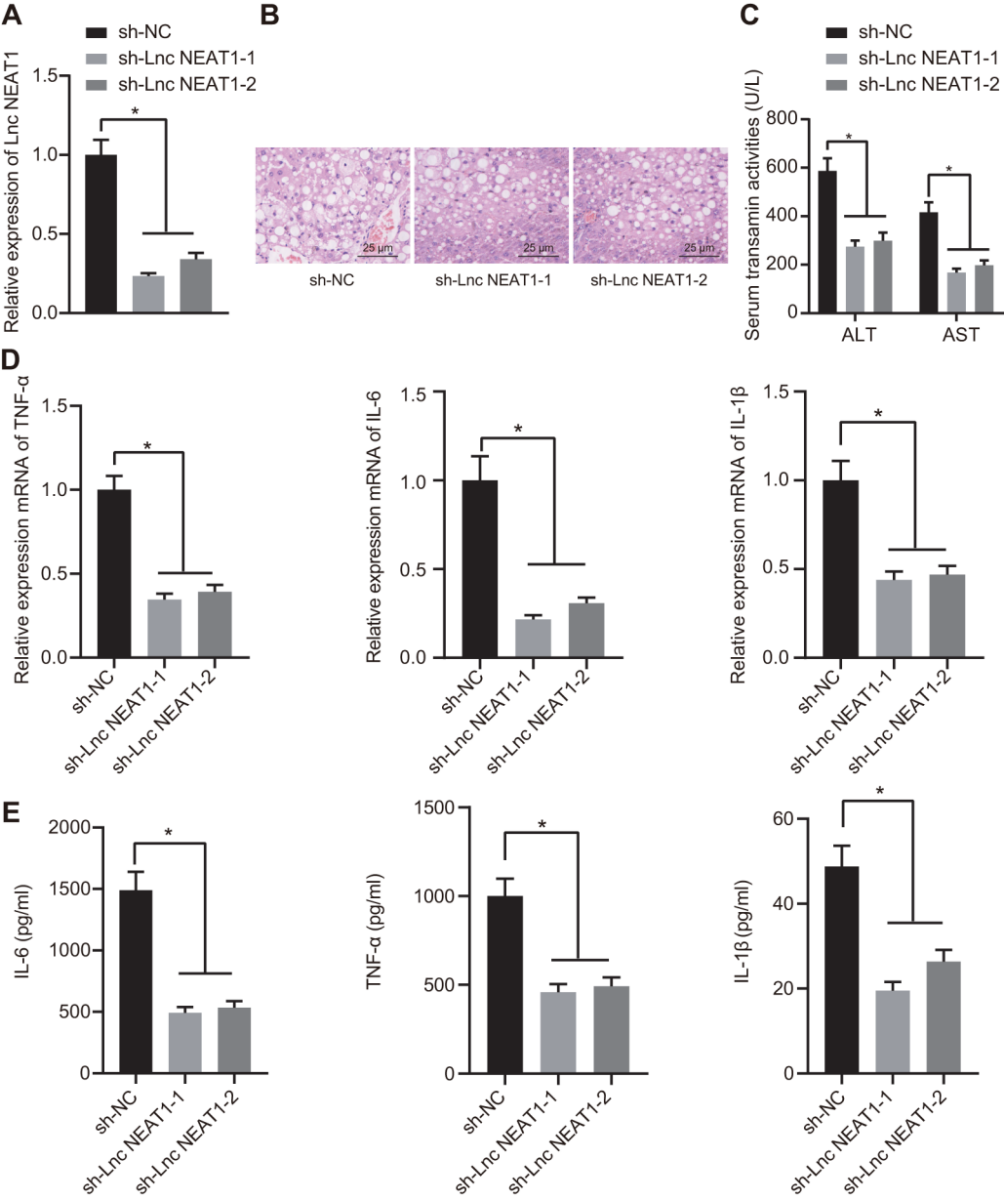
**Silencing NEAT1 alleviated the occurrence of ALF.** (**A**) Results of RT-qPCR for determining the knockdown efficiency of sh-NEAT1. (**B**) H&E staining to study histopathological changes in the liver after silencing NEAT1. (**C**) Biochemical markers ALT and AST activities in ALF mouse serum. (**D**) The mRNA expression levels of TNF-α, IL-6, and IL-1β were determined by RT-qPCR and normalized to sh-NC group. (**E**) ELISA assay to determine the cytokine level in ALF mice. Data were summarized as mean ± S.D. from at least 3 independent biological replicates. One-way ANOVA was applied to compare data among multiple groups. * indicates *p* < 0.05. N = 12 mice for each group.

To further investigate the functional role of lncRNA NEAT1 in ALF, we knocked down lncRNA NEAT1 in ALF mice using adenovirus-mediated transfection of sh-NEAT1. The expression level of lncRNA NEAT1 was decreased by sh-NEAT1 (Figure 3A), validating the knockdown effect. With the knockdown of lncRNA NEAT1, hepatocyte necrosis and apoptosis were alleviated, as indicated by histopathological analysis using H&E staining (Figure 3B). The treatment with sh-NEAT1 also reduced the activity of ALT and AST in the ALF mouse model (Figure 3C). Silencing lncRNA NEAT1 also significantly downregulated the mRNA levels of TNF-α, IL-6, and IL-1β in the liver tissues (Figure 3D) and cytokine levels in the serum (Figure 3E) of ALF mice. In summary, these results demonstrated that lncRNA NEAT1 plays a vital role in ALF as silencing of lncRNA NEAT1 alleviates the development of ALF.

### LncRNA NEAT1 suppresses expression of miR-139 *via* EZH2 and induces inflammatory cytokine production

We sought to clarify the mechanisms underlying the regulation of lncRNA NEAT1 in ALF and explore whether lncRNA NEAT1 suppressed miR-139 through EZH2 in ALF. It was shown that the mRNA levels of EZH2 were increased while miR-139 was decreased in serum samples of patients with ALF, ALF mice, and LPS-treated PBMCs (Figure 4A). Moreover, Western blot analysis showed that the protein levels of EZH2 were upregulated in both ALF mice liver and LPS-treated PBMCs (Figure 4B). Furthermore, the reduction of miR-139 in ALF mice and LPS-induced PBMCs was restored by sh-NEAT1, while overexpressed lncRNA NEAT1 induced the opposing results (Figure 4C), indicating the suppression of lncRNA NEAT1 on miR-139. RIP and ChIP results in LPS-treated PBMCs verified that the binding of EZH2 to lncRNA NEAT1 was decreased (Figure 4D) while the enrichment of H3K27me3 on the promoter region of miR-139 was decreased upon silencing lncRNA NEAT1, yet increased after overexpressing lncRNA NEAT1 (Figure 4E). These data demonstrated that lncRNA NEAT1 recruits EZH2 to promote the enrichment of H3K27me3 on the promoter region of miR-139, leading to the suppression of miR-139.

**Figure 4A-D:**
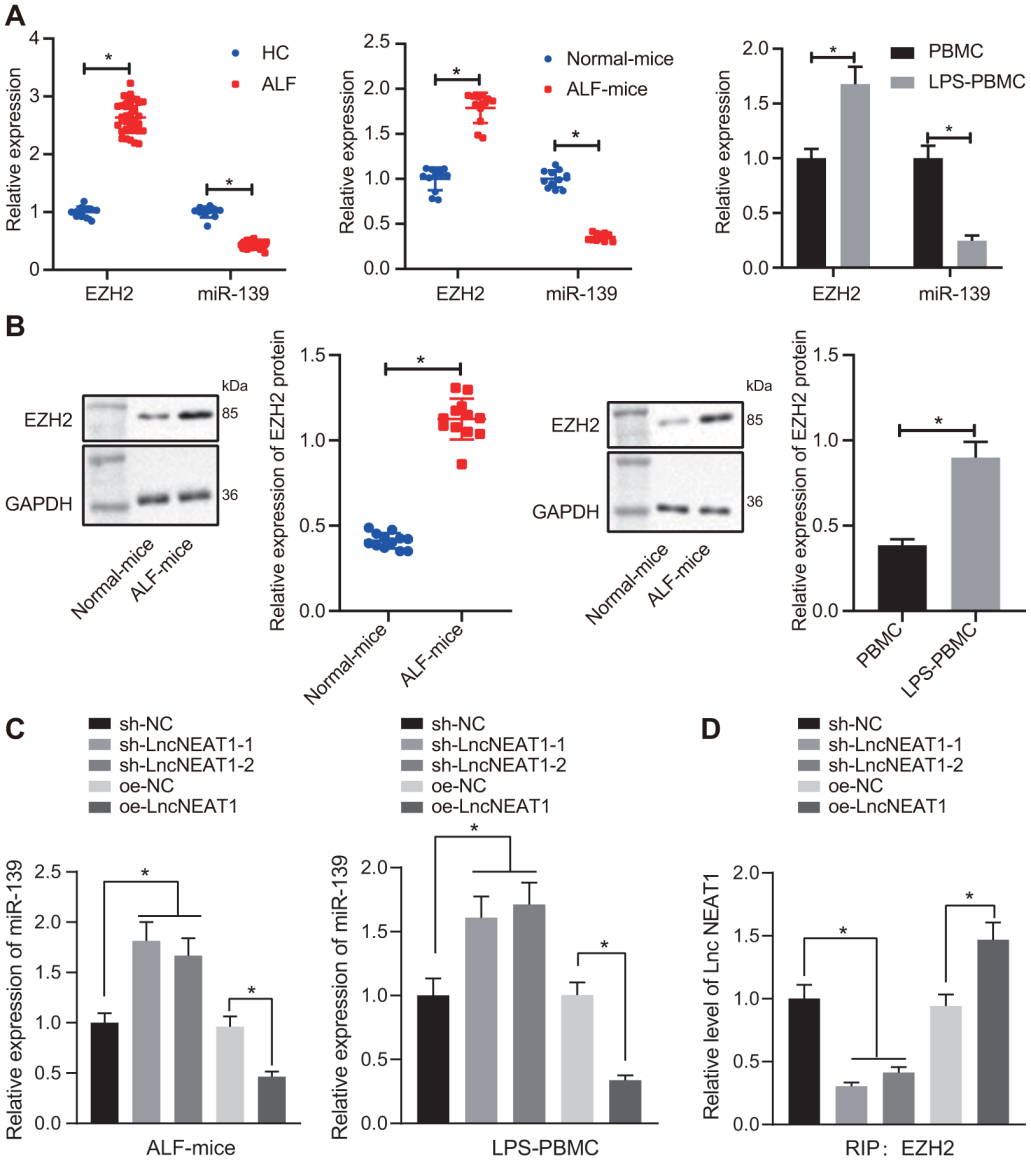
**LncRNA NEAT1 suppressed the expression of miR-139 *via* EZH2 and induced cytokine production.** (**A**) RT-qPCR to determine the expression levels of miR-139 and EZH2 in serum samples of patients with ALF, ALF murine liver tissues, and LPS-induced PBMCs. (**B**) Western blot analysis results to detect the protein levels of EZH2 in ALF murine liver tissues, and LPS-induced PBMCs. (**C**) Expression of miR-139 upon knockdown and overexpression of lncRNA NEAT in LPS-treated PBMCs. (**D**) Interaction between EZH2 and NEAT1 as detected by RIP assay.

**Figure 4E-I:**
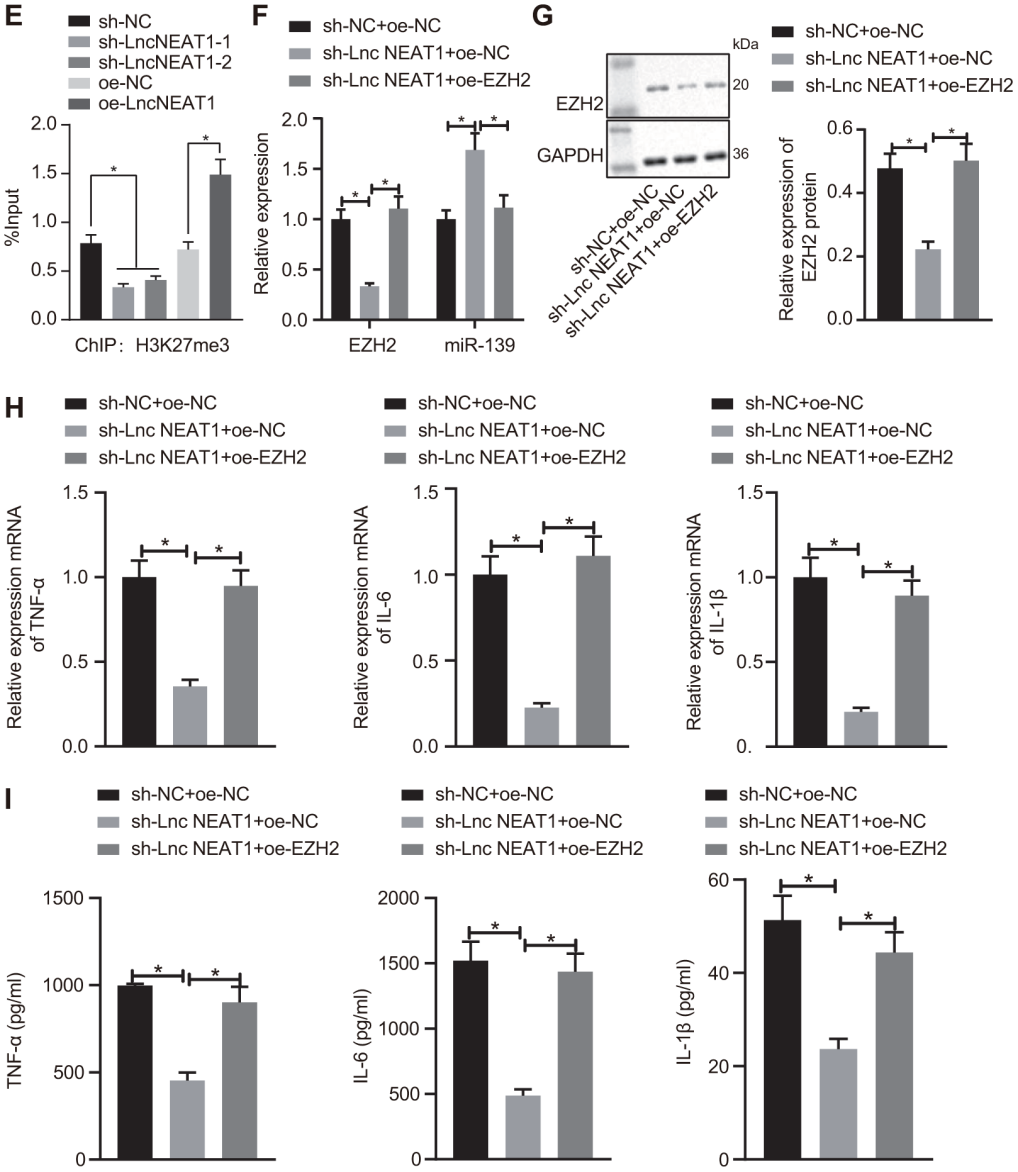
**LncRNA NEAT1 suppressed the expression of miR-139 *via* EZH2 and induced cytokine production.** (**E**) ChIP analysis to determine the enrichment level of H3K27me3 on the promoter region of miR-139 upon knockdown and overexpression of lncRNA NEAT. (**F**) RT-qPCR to determine the expression levels of miR-139 and EZH2 in LPS-treated PBMCs in the presence of sh-NEAT1 with or without oe-EZH2. (**G**) Western blot analysis to determine the protein levels of EZH2. (**H**) mRNA levels of pro-inflammatory cytokines as detected by RT-qPCR. (**I**) ELISA analysis of cytokines levels. Data were summarized as mean ± S.D. from at least 3 independent biological replicates. (**A**, **B**) Data were compared using unpaired student’s *t*-test. (**C**–**I**) One-way ANOVA with Tukey’s post hoc test was applied to compare data among multiple groups. * indicates *p* < 0.05.

Going further, we investigated whether the overexpression of EZH2 affected the regulation of lncRNA NEAT1 on miR-139. The PBMCs with LPS treatment were co-transfected with sh-NEAT1 and oe-EZH2. Consistent with the previous results, silencing lncRNA NEAT1 reduced the expression of EZH2 and upregulated miR-139 as compared with the sh-NC group (Figure 4F, 4G). The upregulation of miR-139 by sh-NEAT1 was inhibited by the overexpression of EZH2 (Figure 4F, 4G). Furthermore, the mRNA level and protein level of the pro-inflammatory cytokines TNF-α, IL-6, and IL-1β reduced by sh-NEAT1 were both restored by overexpression of EZH2 (Figure 4H, 4I), indicating that increasing EZH2 alleviated inflammation in ALF. Overall, these data suggest that lncRNA NEAT1 inhibits the expression of miR-139 via EZH2, and results in the production of pro-inflammatory cytokines.

### miR-139 suppresses production of pro-inflammatory cytokines by targeting PUMA

After demonstrating the regulatory effects of lncRNA NEAT1 on miR-139 in ALF, we next studied how miR-139 affected the development of ALF. We predicted that PUMA was a downstream target gene of miR-139 by utilizing the TargetScan database (Figure 5A). Analysis of the expression levels of PUMA by RT-qPCR and Western blot analysis showed that PUMA was significantly upregulated in ALF as compared with the control groups (Figure 5B, 5C). We applied dual-luciferase reporter gene assay to verify the relationship between miR-139 and PUMA. It was shown that in PUMA-wt-transfected cells, as compared with the NC mimic-transfected cells, the luciferase activity was significantly decreased after miR-139 mimic treatment. However, there was no difference between the NC mimic and miR-139 mimic when cells were co-transfected with PUMA-Mut (Figure 5D). These results suggested that miR-139 specifically targeted PUMA. To further investigate the impact of miR-139 on PUMA and ALF, we overexpressed miR-139 and PUMA simultaneously. As shown in Figure 5E, 5F, miR-139 mimic increased the miR-139 levels and downregulated the mRNA and protein levels of PUMA. Overexpression of PUMA did not affect miR-139 but restored the PUMA levels (Figure 5E, 5F). RT-qPCR and ELISA analysis verified that miR-139 mimic induced higher expression levels of TNF-α, IL-6, and IL-1β level than NC mimic (Figure 5G, 5H). In association with the oe-PUMA, the miR-139-mediated decrease of pro-inflammatory cytokines was upregulated by oe-PUMA (Figure 5G, 5H). Overall, these data indicated that miR-139 targets PUMA leading to inhibition, succeeded by a suppression of pro-inflammatory cytokine production.

**Figure 5A-D:**
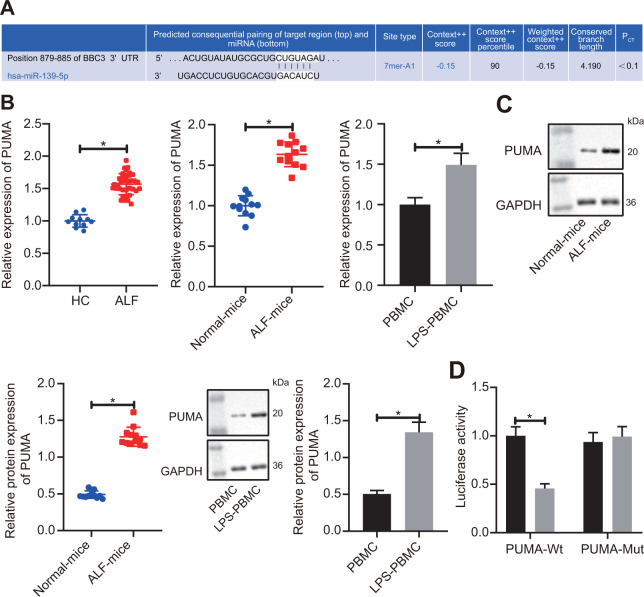
**miR-139 suppressed production of pro-inflammatory cytokines by targeting PUMA.** (**A**) Prediction of binding of miR-139 to PUMA-3’UTR by TargetScan database (http://www.targetscan.org/vert_71/). (**B**) RT-qPCR to determine the expression levels of PUMA in serum samples of patients with ALF, ALF murine liver tissues, and LPS-induced PBMCs. (**C**) Results of Western blot analysis to determine the protein levels of PUMA in ALF murine liver tissues, and LPS-induced PBMCs. (**D**) Dual-luciferase reporter gene assays to analyze the target relationship between miR-139 and PUMA.

**Figure 5E-H:**
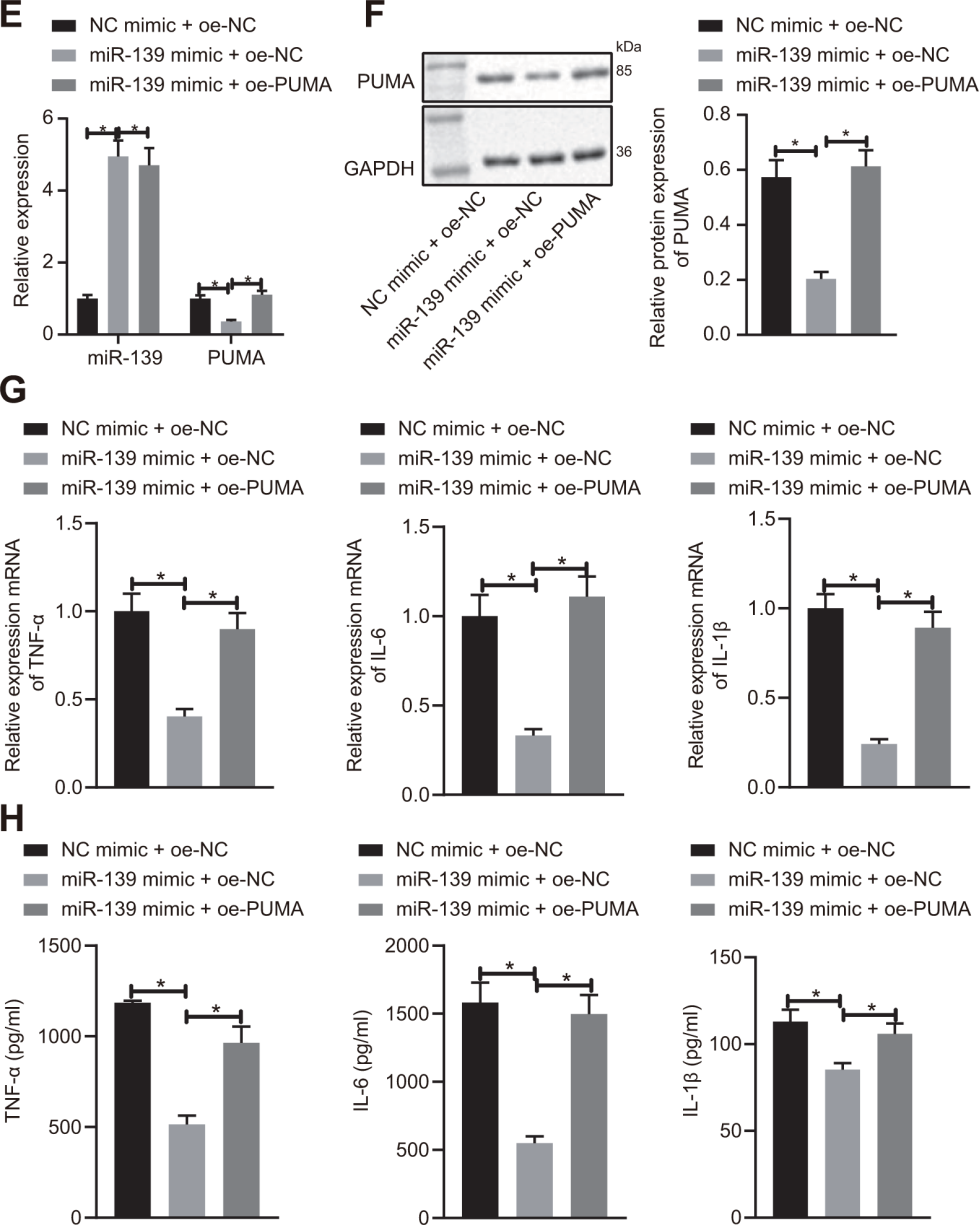
**miR-139 suppressed production of pro-inflammatory cytokines by targeting PUMA.** (**E**, **F**) PUMA mRNA (**E**) and protein (**F**) level in the transfected cells. (**G**) mRNA level of pro-inflammatory cytokines as determined by RT-qPCR. (**H**) ELISA analysis of cytokines levels. Data were summarized as mean ± S.D. from at least 3 independent biological replicates. (**B**–**D**) Data were compared using unpaired student’s *t*-test. (**E**–**H**) One-way ANOVA with Tukey’s post hoc test was applied to compare data among multiple groups. * indicates *p* < 0.05.

### Silencing of lncRNA NEAT1 attenuates the development of ALF through PUMA

**Figure 6:**
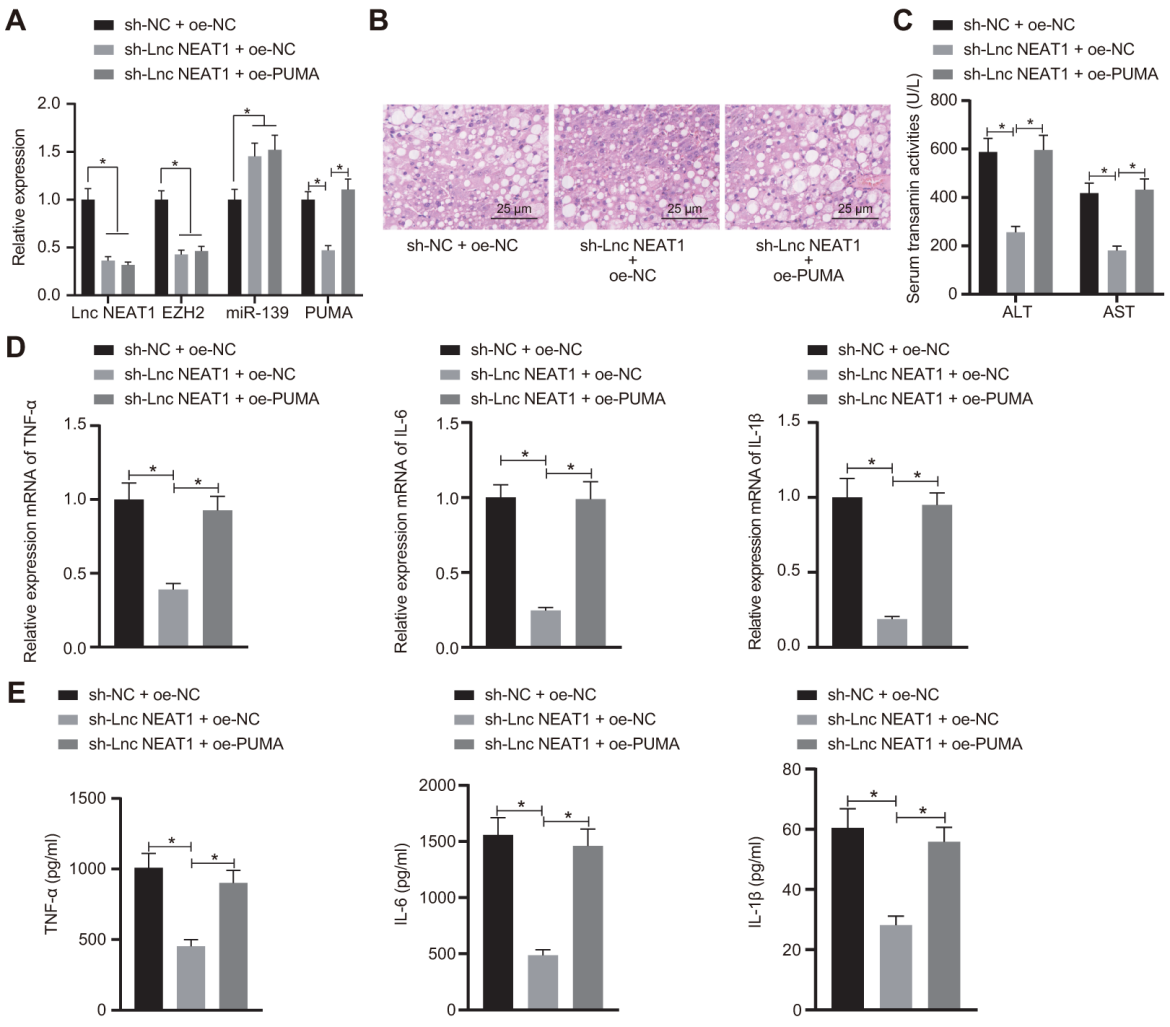
**Silencing of lncRNA NEAT1 attenuates the development of ALF through PUMA.** (**A**) RT-qPCR analysis of lncRNA NEAT1, EZH2, miR-139, and PUMA levels in ALF mice. (**B**) Histological analysis of ALF mouse liver tissues. (**C**) Biochemical analysis of ALT and AST activity in ALF mouse serum samples. (**D**) Pro-inflammatory cytokines TNF-α, IL-6, and IL-1β mRNA level as detected by RT-qPCR. (**E**) ELISA assay performed to detect the TNF-α, IL-6 and IL-1β levels. Data were summarized as mean ± S.D. from at least 3 independent biological replicates. One-way ANOVA with Tukey’s post hoc test was applied to compare data among multiple groups. * indicates *p* < 0.05. n = 12 mice for each group.

We finally sought to determine whether lncRNA NEAT promoted ALF in a PUMA-dependent manner. We injected sh-NEAT1 with or without PUMA and studied the effects in ALF mice. As shown in Figure 6A, the knockdown of lncRNA NEAT1 with sh-NEAT1 significantly decreased the expression levels of lncRNA NEAT1, EZH2, and PUMA. Meanwhile, the overexpression of PUMA had no effects on lncRNA NEAT1, EZH2, and miR-139 but restored the PUMA level (Figure 6A). H&E staining suggested that sh-NEAT1 relieved hepatocyte necrosis and apoptosis, which diminished upon co-treatment with oe-PUMA (Figure 6B). The biochemical analysis also showed that the sh-NEAT1-mediated reduction of ALT and AST activity was upregulated by oe-PUMA (Figure 6C). In addition, the mRNA levels and protein levels of TNF-α, IL-6 and IL-1β were decreased by sh-NEAT1 treatment, but increased by overexpressed PUMA even in the presence of sh-NEAT1 (Figure 6D, 6E). In summary, these data demonstrate that silencing of lncRNA NEAT1 alleviates the development of ALF *via* PUMA.

## DISCUSSION

Intense systemic inflammation is a feature of ALF, which can result in organ failure [24]. ALF is reported to be an inflammation-regulated disease [25]. Thus, the exploration of molecular mechanisms underlying inflammation in ALF may provide potential targets for ALF prevention. Our current data indicated that lncRNA NEAT1, EZH2, miR-139 and PUMA were implicated in the inflammatory mechanisms of ALF.

Prior to the analysis, the ALF rodent model induced by D-GalN/LPS was established, in which ALT and AST were highly expressed. High expression of ALT and AST suggests the successful development of ALF and low survival rate *in vivo* [26]. Specifically, the role of lncRNA NEAT1 in ALF was analyzed in the present study. The roles of lncRNAs in the inflammation of liver injury have been widely studied. As observed in previous work, 9 lncRNAs had high expression in ALF, of which 2 lncRNAs are related to monocarboxylate transporter 2 and neuroepithelial transforming gene 1, suggesting that lncRNAs may exert certain function in inflammation-mediated ALF [27]. It is indicated that the expression of lncRNA NEAT1 can be upregulated by LPS treatment [28], which is similar to our results derived from the D-GalN/LPS-induced ALF model. A previous study also demonstrated that lncRNA NEAT1 promotes the development of LPS-induced liver injury by stimulating inflammatory responses [11], which is in line with our results showing that lncRNA NEAT1 was highly expressed in ALF. Furthermore, we found that silencing lncRNA NEAT1 was able to alleviate ALF by reducing the expression of inflammatory factors TNF-α, IL-6 and IL-1β. TNF-α, IL-6 and IL-1β are typically recognized pro-inflammatory cytokines in hepatic injuries [29]. It is illustrated that the pro-inflammatory cytokines TNF-α, IL-6 and IL-1β are upregulated in ALF [30]. As the evidence collected in our study indicated, knockdown of lncRNA NEAT1 reduced TNF-α, IL-6, and IL-1β expression to suppress the inflammatory response, thereby alleviating ALF.

Subsequently, we analyzed the downstream regulatory mechanism underlying lncRNA NEAT1, in which we found that lncRNA NEAT1 regulated miR-139 expression through EZH2. Highly expressed EZH2 and H3K27me3 can contribute to the inflammatory response in ALF through the TNF pathway [31]. It has been reported that lncRNA NEAT1 binds to the histone methyltransferase EZH2 and subsequently inhibits the expression of the downstream target genes in glioblastoma [13]. Moreover, EZH2 is able to upregulate the expression of miR-139 by promoting the tri-methylation of H2K27 in pancreatic cancer cells [14]. The data derived from our study suggested that lncRNA NEAT1 decreased miR-139 expression by promoting EZH2. In nonalcoholic fatty liver disease, miR-139-5p expression is decreased, which can reduce the expression of inflammatory cytokines [32]. The implication of inflammation has been highlighted in responses to damage, including cognitive performance [33]. miR-139 is specifically related to inflammatory regulation in hepatic failure [34]. In our study, we proved that overexpressed miR-139 effectively lowered the expression levels of TNF-α, IL-6, and IL-1β. The bioinformatics website TargetScan was also employed in our study to determine the potential binding sites between miR-139 and PUMA, showing that miR-139 targeted PUMA, which, for the first time demonstrated the regulatory effect of miR-139 on PUMA, as revealed by the dual-luciferase reporter gene assay. PUMA is shown as a pro-apoptotic factor [35]. Inhibition of PUMA is found to alleviate ALF in modeled mice [36], which is consistent with our finding that downregulation of PUMA, regulated by miR-139, alleviated ALF by weakening the inflammatory response.

## CONCLUSIONS

Taking these findings together, the evidence presented in our study demonstrated an inflammation regulatory mechanism where silencing of lncRNA NEAT1 upregulated miR-139 through EZH2, which, in turn, diminished PUMA expression and restricted inflammatory response, thus alleviating the progression of ALF. These results validated the role of an lncRNA NEAT1-dependent mechanism in the inflammation of ALF and propose a novel therapeutic molecular target for ALF. However, these findings are preliminary and further investigation into the comprehensive mechanism of action is warranted.

## MATERIALS AND METHODS

### Human patient samples

Human serum samples were collected from 35 patients with ALF (23 males, 12 females, mean age: 41.17 ± 9.43 years old) who were diagnosed and treated in the Transplantation Center in The Third Xiangya Hospital of Central South University from January 2017 to January 2019. The diagnosis of ALF was based on the criteria of Guideline for Diagnosis and Treatment of Liver Failure (2006 edition). ALF was defined as severe liver injury by hepatic encephalopathy, where the onset of encephalopathy was within 8 weeks ever since the first symptoms of illness, without the occurrence of any pre-existing liver disease [37]. Eleven healthy subjects with a mean age of 39.91 ± 8.35 years old were recruited as healthy control (HC), including 6 males and 5 females.

### ALF mouse model

C57BL/6 female mice (SLAC Laboratory Animal Co., Ltd., Shanghai, China) aged ten weeks and weighing 18 - 20 g were maintained in a sterile environment at a temperature of (22 – 25)° C with a 12-h/12-h light-dark cycle. Mice were fed rodent chow diet and water *ad libitum* for at least 2 weeks, followed by fasting for 12 h before experiments. To generate the ALF mouse model, mice (12 mice for each treatment) received 10 μg/kg of LPS (Sigma-Aldrich, St. Louis, MO, USA) and 700 mg/kg of D-galactosamine (D-GalN, Sigma-Aldrich) in 200 μL of sterile phosphate buffer saline (PBS) solution intraperitoneally (i.p.) for 6 h [31]. For the experimental control, mice (n = 12) received an equal volume of PBS i.p. administration.

Prior to the ALF modeling, mice were i.p. injected with Adenoviral-based short hairpin RNA targeting lncRNA NEAT1 (sh-NEAT1), PUMA overexpression vector (oe-PUMA) or the corresponding negative control (NC) to interfere with the expression of lncRNA NEAT1 and PUMA. The mice were anesthetized and euthanized by cervical dislocation, 6 h after modeling. Liver and serum samples were harvested for analysis. The 12-h survival rate, liver histology, and biochemical indicators were analyzed to verify the successful induction of the ALF murine model.

### PBMC isolation and stimulation

Human PBMCs were prepared using the Ficoll density gradient centrifugation method as previously described [31]. Isolated PBMCs were maintained in Roswell Park Memorial Institute 1604 medium (Gibco, Gaitherburg, MD, USA) containing 10% fetal bovine serum (FBS) (Excell Bio, Shanghai, China), and cultured at 37° C in 5% CO_2_. Cells were treated with plasmids of sh-NEAT1, EZH2 overexpression vector (oe-EZH2), miR-139 mimic, oe-PUMA or the corresponding NC using lipofectamine 2000 reagent (Invitrogen, Carlsbad, CA, USA) to either silence or overexpress the target genes. To induce an inflammatory response, the cells were treated with 1 μg/mL of LPS post transfection.

### Detection of biochemical indicators

Mouse serum samples were collected and analyzed using a Hitachi 7170 Chemistry Analyzer (Hitachi, Tokyo, Japan). Activities of ALT and AST were obtained as indicators of the liver function.

### Histological analysis

Liver tissues were fixed with 10% formalin. Paraffin-embedded sections were subjected to Hematoxylin and Eosin (H&E) staining [30]. Morphology of the liver sections was then observed and images were captured using a microscope (Axio Observer A1/D1/Z1, ZEISS, Oberkochen, Germany).

### Reverse transcription quantitative polymerase chain reaction (RT-qPCR)

Tissues and cells were homogenized using TRIzol (Invitrogen) to extract RNA. The concentration and purity of the extracted RNA were determined using a Nanodrop 2000 (1011U, Thermo Fisher Scientific, MA, USA). For reverse transcription, TaqMan MicroRNA Assays Reverse Transcription primer (Applied Biosystems, MA, USA) and PrimeScript RT reagent Kit (Takara, Shiga, Japan) were used, following the manufactures’ instructions. Primers for lncRNA NEAT1, miR-139, tumor necrosis factor alpha (TNF-α), interleukin 6 (IL-6), and IL-1β were synthesized by Takara Bio Inc (Tokyo, Japan) (Table 1). Relative quantitation of RNA was assessed by RT-qPCR and analyzed using the comparative quantitation method (2^-ΔΔCt^). Glyceraldehyde 3-phosphate dehydrogenase (GAPDH) served as a reference gene for mRNA and U6 was served as a reference gene for miR-139.

**Table 1:** Primer sequences for RT-qPCR.

**Gene**	**Sequences**
*NEAT1*	F: 5’-GTAATTTTCGCTCGGCCTGG-3’
	R: 5’-TACCCGAGACTACTTCCCCA-3’
*miR-139*	F: 5’-CCCAAAGACAAGCAGGACTC-3’
	R: 5’-TGGGGTAGACTAAGGCCAGA-3’
*TNF-α*	F: 5’-CAACGCCCTCCTGGCCAACG-3’
	R: 5’-TCGGGGCAGCCTTGTCCCTT-3’
*Il-6*	F: 5’-TAGTCCTTCCTACCCCAATTTCC-3’
	R: 5’-TTGGTCCTTAGCCACTCCTTC-3’
*Il-1β*	F: 5’-AGAGCATCCAGCTTCAAATCTC-3’
	R: 5’-CAGTTGTCTAATGGGAACGTCA-3’
*U6*	F: 5’-TGCGGGTGCTCGCTTCGGCAGC-3’
	R: 5’-TGGGGTAGACTAAGGCCAGA-3’
*GAPDH*	F: 5’-GCACCGTCAAGGCTGAGAAC-3’
	R: 5’-TGGTGAAGACGCCAGTGGA-3’

Notes: RT-qPCR, Reverse transcription quantitative polymerase chain reaction; F, forward; R, reverse; *NEAT1,* nuclear enriched abundant transcript 1; *miR-139*, microRNA 139; *TNF-α*, tumor necrosis factor alpha; *Il-6*, interleukin 6; *Il-1β*, interleukin 1 beta; *U6*, U6 small nuclear RNA; *GAPDH*, glyceraldehyde 3-phosphate dehydrogenase.

### Western blot analysis

Total protein samples were extracted from tissues or cells using Radio immunoprecipitation (RIPA) lysis buffer (Beyotime Biotechnology, Shanghai, China) containing phenylmethylsulfonyl fluoride. The total protein concentration was determined using a Pierce™ BCA Protein Assay Kit (Thermo Fisher Scientific). Fifty micrograms of protein were subjected to standard sodium dodecyl sulfate–polyacrylamide gel electrophoresis and transferred onto a polyvinylidene fluoride (PVDF) membrane, followed by incubation with primary antibodies (Abcam Inc., Cambridge, UK) EZH2 (ab186006), PUMA (ab9643) and GAPDH (ab9485) at 4° C overnight. After incubation with the horseradish peroxidase (HRP)-conjugated secondary antibody (Abcam), the protein signal was developed with Enhanced chemiluminescence (ECL) Immunoblot Detection Kit (Amersham, UK) and analyzed with Bio-Rad imaging systems (Bio-Rad Laboratories, CA, USA). The images were analyzed using Quantity One V4.6.2 and the relative protein quantity was determined by normalizing to GAPDH, as represented by the gray levels.

### Enzyme-linked immunosorbent assay (ELISA)

Serum samples and cell culture supernatant were subjected to ELISA assays to measure pro-inflammatory cytokines. Following the ELISA kit manufacturer’s instructions, the contents of TNF-α (RAB0477, Sigma-Aldrich), IL-6 (RAB0308, Sigma-Aldrich) and IL-1β (RAB0274, Sigma-Aldrich) were determined.

### Dual-luciferase reporter gene assay

PUMA was predicted as the downstream target gene of miR-139 using the TargetScan database (http://www.targetscan.org/vert_71/). The miR-139 binding region of wild type (wt) PUMA 3’-UTR-recombined pmirGLO vector (*pmirGLO-PUMA-wt*) and point mutation PUMA pmirGLO vector (*pmirGLO-PUMA-mut)* vector was synthesized and sequenced by RiboBio Co., Ltd. (Shanghai, China). The *pmirGLO-PUMA-wt* or *pmirGLO-PUMA-mut* was co-transfected with miR-139 mimic or NC mimic into HEK293T cells, and the cells were subsequently cultured in Dulbecco Modified Eagle Medium/10% FBS for 48 h. Next, 100 μL of cell lysate was mixed with the detection solution to detect the renilla luciferase and firefly luciferase activity levels using a microplate reader (SpectraMax M5, Molecular Devices, CA, USA).

### RNA immunoprecipitation (RIP) assay

Magna RIP™ RNA-Binding Protein Immunoprecipitation Kit (Millipore, Darmstadt, Germany) was applied to detect the binding between lncRNA NEAT1 and EZH2, following the methods described in the user guides. In brief, cells were homogenized with RIPA lysis buffer (Beyotime) and centrifuged at 12,000 ×g at 4° C for 10 min. The supernatant was collected and incubated with magnetic bead-EZH2 antibody (ab186006, 1: 100, Abcam) complex or magnetic bead-Immunoglobulin G (IgG) (ab172730, Abcam) overnight at 4° C. Binding RNA was purified by magnetic separation and Proteinase K detachment, followed by RT-qPCR.

### Chromatin immunoprecipitation (ChIP) assay

Cells at 70% - 80% confluence were fixed with 1% formaldehyde for 10 min at room temperature. DNA-protein cross-linkage was achieved and ultrasonic treatment was applied to the cell lysates. Digested chromatin was collected in the supernatant by centrifugation at 13,000 ×g at 4° C, and subsequently incubated with protein-specific rabbit antibody to H3K27me3 (ab192985, 1 : 100, Abcam) or IgG as NC (ab172730, Abcam) overnight at 4° C. The DNA-protein complex was precipitated by Protein Agarose/Sepharose and de-crosslinked overnight at 65° C. The DNA fragment was purified by phenol/chloroform extraction method, followed by RT-qPCR to detect the enrichment of H3K27me3 on the promoter region of miR-139.

### Statistical analysis

Data were summarized as mean ± standard deviation (S.D.) from at least 3 independent biological replicates. Data analysis was performed using SPSS 19.0 (IBM SPSS Statistics, Armonk, NY, USA). Data represented the average of three experiments performed in triplicates. Comparison of data with normal distribution and equal variance between two groups was done using unpaired student’s *t*-test. Comparison between multiple groups was done using one-way analysis of variance (ANOVA) with Tukey’s post hoc test. Differences were considered statistically significant if *p* < 0.05.

### Ethics statement

Written informed consent was obtained from all patients and all study procedures were compliant with the Declaration of Helsinki. The study protocol was approved by the Ethics Committee of The Third Xiangya Hospital of Central South University. The animal experiments were carried out after approval of the Animal Ethics Committee of The Third Xiangya Hospital of Central South University and were in accordance with the Guide for the Care and Use of Laboratory Animals published by the National Institutes of Health. All care was taken to minimize the number and suffering of animals included in the study.
